# Comparison of primary care prescriptions for old and very old hypertensive patients Selcan

**DOI:** 10.55730/1300-0144.5618

**Published:** 2023-02-02

**Authors:** TÜLÜ ÇOLAK, Caner VIZDIKLAR, Mert KAŞKAL, Volkan AYDIN, Ömer ATAÇ, Ahmet AKICI

**Affiliations:** 1 Department of Medical Pharmacology, School of Medicine, Marmara University, İstanbul, Turkey; 2 Department of Medical Pharmacology, International School of Medicine, İstanbul Medipol University, İstanbul, Turkey; 3 Marmara University Hypertension and Atherosclerosis Research Center (HİPAM), İstanbul, Turkey; 4 Department of Public Health, International School of Medicine, İstanbul Medipol University, İstanbul, Turkey; 5 Department of Health Management and Policy, College of Public Health, University of Kentucky, Lexington, United States

**Keywords:** Elderly, family physician, irrational prescribing, hypertension

## Abstract

**Background/aim:**

Elderly and very elderly individuals might be subject to different approaches for the treatment of hypertension. We aimed to compare drug utilization in hypertensive old patients and very old patients in primary care, along with the evaluation of potentially inappropriate drug prescribing.

**Materials and methods:**

In this cross-sectional study, we compared prescriptions of 65–79-year-old (old patient prescriptions [OPP], n = 433,988) vs. ≥80-year-old (very old patient prescriptions [VOPP], n = 134,079) with “essential hypertension” diagnosis, issued by 3:1 systematically-sampled primary care physicians (n = 1431) in İstanbul throughout 2016. Drug utilization patterns and distribution of antihypertensives based on drug class and combination status were evaluated. Frequency of potentially inappropriate drugs per Beers Criteria were identified and compared.

**Results:**

Antihypertensive monotherapy practice was less common in OPP than VOPP (43.3% vs. 45.3%; p < 0.001). In both groups, the most commonly prescribed drugs were beta-blockers for monotherapy (37.4% vs. 33.1%, p < 0.001) and thiazide diuretics for combined therapy (69.8% vs. 67.4%, p < 0.001). Metoprolol was the most commonly prescribed antihypertensive both in OPP and VOPP (15.3% vs. 14.8%). Furosemide was ranked 10th in OPP and 3rd in VOPP (2.7% vs. 5.5%). Cardiovascular system drugs were the most commonly encountered potentially inappropriate medications in both groups (263.9 vs. 283.4 per 10,000 prescriptions, p = 0.004). Regarding antihypertensive drugs, 2.2% of those in OPP and 2.4% of those in VOPP were identified as potentially inappropriate (p = 0.002).

**Conclusion:**

Prescribing preferences to old and very old patients mostly showed slight differences. Almost half of prescriptions comprising antihypertensive monotherapy might imply hesitancy to prescribe combinations. Overuse of risky drugs such as furosemide in both groups, especially in the very elderly, requires more attention.

## 1. Introduction

Hypertension is the most common chronic condition in the elderly with well-recognized outcomes such as cardiovascular morbidity and mortality [1,2]. Although hypertension demonstrates higher prevalence in advanced ages, those individuals might also be at risk of hypotension associated with frailty and impaired baroreflex sensitivity [1,3]. Thus, blood pressure goals in very old patients (i.e. over 80) were set as less strict, and the recent guidelines recommended a less aggressive approach for initiation of pharmacotherapy for those patients [3,4].

Older individuals might be at higher risk of polypharmacy due to higher number of comorbidities and related drug treatment burden. Aside from potential issues such as drug interactions, altered drug elimination and intolerance to adverse events, several drugs may interfere with the treatment of hypertension, interrupting control, and provoking any complications [1,5]. Also, very old patients could be at higher risk for those outcomes due to higher prevalence of comorbidities and potential frailty [6]. Thus, rational management of the increasing burden of drugs for older hypertensive patients is of considerable importance. Guidelines on potentially inappropriate medications (PIMs) such as Beers Criteria might be utilized to detect and prevent any drug-related issues in the elderly [7].

Primary care plays a key role in the diagnosis, treatment, and follow-up of hypertension. High number of comorbidities and related burden of treatment in geriatric population might complicate the management of such chronic diseases in these centers [8]. In this regard, rational use of antihypertensives in “old patients” and “very old patients”, which might be subject to different approaches in the treatment of hypertension, might contribute to reducing the burden of pharmacotherapy brought on by either hypertension or other comorbidities. This study aimed to compare drug utilization in hypertensive “old patients” and “very old patients” in primary care, along with the evaluation of PIM prescribing in these groups.

## 2. Materials and methods

In this cross-sectional descriptive study, prescriptions issued in primary care centers of İstanbul in 2016 were examined retrospectively. Prior to data collection, ethical approval was obtained from İstanbul Medipol University, Non-Interventional Clinical Studies Ethics Committee (date: 14.10.2021, approval number: 1007).

İstanbul was home to 14.8 million inhabitants in 2016, which comprised 18.5% of the population of Turkey.^1^ Out of 4293 primary care units active in 2016 midyear, the minimum number of units to achieve adequate sample size was calculated as 353 at 95% confidence level, 5% margin of error, and 50% prevalence. Using systematic sampling, 1431 primary care units with a physician were selected. The dataset included the prescriptions generated in those units between January 1 and December 31, 2016 and registered to national Prescription Information System [9]. Prescriptions for patients ≥65 years of age (n = 1,384,255) were identified and those with “I10-essential (primary) hypertension” diagnosis (n = 568,301) according to International Classification of Diseases-10 (ICD-10) were selected. Due to the potential errors of age value typing/registration, those written to ≥110 years (n = 234) were excluded, and the remaining 568,067 prescriptions were included in the study (Figure).

Prescriptions were split into two based on age definitions in European Society of Cardiology/European Society of Hypertension (ESC/ESH) guidelines, as those generated for individuals aged 65–79 years as “old patient prescriptions” (OPP, n = 433,988) and ≥80 years as “very old patient prescriptions” (VOPP, n = 134,079) [3]. Mean ages, sex distribution, and concomitant diagnoses of the recipients, drugs included in the prescriptions, and drug parameters per prescription were examined and compared. The most commonly prescribed 30 antihypertensive drugs in each group were determined. In addition, distribution of antihypertensives based on class, and monotherapy/sole single-pill combination (SPC)/multiple drug combination (free combination or multiple SPCs) presence in prescriptions containing antihypertensive drugs were evaluated and compared. Furthermore, 2015 Beers Criteria, which was the most recent at the time of the study, were used to identify and compare the frequency of PIMs in both groups. Drugs listed as “medications to avoid” in Beers Criteria were defined as PIM, and prescriptions including at least one of those medications were identified accordingly [7]. In addition, drugs that were listed in Beers Criteria, yet of which potential inappropriateness could not be fully evaluated by follow-up, were presented separately.

### 2. 1. Statistical analysis

Data were analyzed using IBM SPSS Statistics 22.0 (IBM Corp., Armonk, NY, USA) and GraphPad Prism 5.0 (GraphPad Software, San Diego, CA, USA) software. Analyzed data were expressed as numbers, percentages, and/or mean ± standard deviation values, where appropriate. Frequency analysis was used for statistical evaluation, while categorical variables were compared by chi-square test. Normality of distribution for continuous variables was evaluated using the Kolmogorov–Smirnov analysis. Normally distributed data were compared using Student’s *t*-test, and for else, the Mann–Whitney U test was used. In order to infer statistical significance, an overall 5% type-I error level was assumed as acceptable.

## 3. Results

We identified that 61.8% of all prescriptions evaluated were issued to women, and the mean age of recipients was 74.4 ± 7.1 years. In total, 89.7% of the prescriptions included one or more antihypertensive drugs. The number of antihypertensives per prescription was 1.2 ± 0.7 (Table 1).

About 76.4% of the prescriptions were OPP, while the remaining 23.6% were issued as VOPP. Female predominance was higher in VOPP (p < 0.001). Less people in OPP than VOPP used monotherapy as antihypertensive regimen (43.3% vs. 45.3%, respectively; p < 0.001). Preference for sole SPC was more frequent in OPP (30.5% vs. 26.6%, p < 0.001), (Table 1).

“Diseases of esophagus, stomach, and duodenum”, “diabetes mellitus”, and “soft tissue disorders” were among the top three comorbidities in both groups. Cardiovascular diseases constituted 8.1% of concomitant diagnoses in OPP, including “ischemic heart diseases”, which was ranked fifth and accounted for 4.5%. In VOPP, cardiovascular disorders comprised 9.7% of concurrent diagnoses, with “ischemic heart diseases” ranking fourth and making up 4.7% of all diagnoses (Table S1). The most commonly prescribed drugs except antihypertensives were acetylsalicylic acid (ASA), metformin, and diclofenac in OPP; and ASA, diclofenac, and pantoprazole in VOPP (Table S2).

Antihypertensive drugs accounted for 31.6% (n = 511,963) and 30.4% (n = 160,300) of all drugs in OPP and VOPP, respectively. The top two antihypertensives in both groups were metoprolol (15.3% vs. 14.8%) and amlodipine (9.5% vs. 10.0%), whereas the third drug was valsartan/hydrochlorothiazide in OPP (5.4%) and furosemide in VOPP (5.5%); with the latter being 10th in OPP (2.7%, Table 2).

The top antihypertensive class preferred for monotherapy was beta-blockers in both OPP and VOPP (37.4% vs. 33.1%, p < 0.001). Calcium channel blockers (CCBs) and angiotensin-converting enzyme inhibitors (ACEIs) were second and third (p < 0.001 for each). The most commonly prescribed antihypertensive class in combination therapy was thiazide diuretics (69.8% in OPP vs. 67.4% in VOPP, p < 0.001). These were followed by angiotensin receptor blockers (ARBs) and ACEIs (p < 0.001 for each). ARBs were present in 2.2% of ACEI prescriptions in OPP and 2.5% of those in VOPP (Table 3).

PIMs constituted 2.0% of all drugs prescribed, as 1.8% of drugs in OPP and 2.4% of drugs in VOPP were potentially inappropriate (p < 0.001). We identified at least one PIM in 6.6% of OPP and 8.8% of VOPP (p < 0.001), with multiple PIMs encountered in 0.3% of OPP and 0.5% of VOPP (p < 0.001). Cardiovascular system drugs were the top PIM class per 10,000 prescriptions in both OPP and VOPP (263.9 vs. 283.4). Share of cardiovascular PIMs were higher in VOPP (p = 0.004), (Table 4). Antihypertensives constituted all of the cardiovascular PIMs (n = 15,255). Antihypertensive PIMs, including doxazosin (n = 14,712), terazosin (n = 243), methyldopa (n = 217), reserpine (n = 71), and immediate release preparations of nifedipine (n = 12) represented 2.3% of all antihypertensives. About 2.2% of antihypertensives in OPP and 2.4% of those in VOPP were potentially inappropriate (p = 0.002). Cardiovascular drugs of which inappropriateness could not be fully evaluated consisted of digoxin (n = 3451) and amiodarone (n = 1745). In addition, proton-pump inhibitors (PPIs) and nonsteroidal antiinflammatory drugs (NSAIDs) were commonly encountered in both groups (1776.4 vs. 1904.7 and 1262.9 vs. 1150.2 per 10,000 prescriptions, respectively), with PPIs being more commonly encountered in VOPP and NSAIDs appearing more frequently in OPP (p < 0.001), (Table 5).

## 4. Discussion

This study assessed more than 500,000 prescriptions with hypertension diagnosis issued for elderly from different age groups, and established substantial findings about the distribution of antihypertensives, drug class preferences of physicians in monotherapy/combined therapy, comorbidities of the recipients, and other prescribing-related details. We identified that more than half of antihypertensive prescriptions contained antihypertensive drug combinations. Preferences of physicians regarding antihypertensive classes varied in monotherapy and combined therapy regimens, yet deviations in frequencies of each in OPP and VOPP were limited. Additionally, it was noteworthy that various drugs such as furosemide or those considered potentially inappropriate were prescribed considerably frequently to geriatric hypertensive patients, especially to the potentially frailer very old.

Consistent with a nationwide study reporting higher prevalence of hypertension in women over 65 (approximately three-fourths vs. three-fifths in men), more prescriptions were written to women in both groups [10]. Remarkably, sex gap was higher in VOPP, which might be attributed to longer life expectancy in women.^2^ It could be suggested that the observed disparity in sex distribution of the groups could possibly influence the distribution of comorbidities and related drug utilization to an extent. Nevertheless, the most common concomitant diagnoses in OPP and VOPP mainly appeared to be comparable, minimizing the likelihood of any discernible difference resulting from sex disparity in that aspect.

Prescriptions with antihypertensive monotherapy regimen were slightly more common in VOPP (45.3% vs. 43.3% in OPP). Due to the limited success of monotherapy in majority of patients, combining antihypertensives were recommended for most groups to achieve desired blood pressure levels [4]. By contrast, the most recent ESC/ESH guidelines which were published after the study period refrained from any aggressive pharmaceutical recommendations for very old or frailer patients, stating monotherapy might be contemplated among first-line choices for these individuals [3]. From that perspective, while monotherapy preference seems reasonable in VOPP, this choice being encountered in nearly half of OPP might imply possible hesitancy to initiate drug combinations even if needed. This might potentially lead to inadequate blood pressure control in some individuals and needs further studies with blood pressure monitoring data for confirmation. In addition, considerably high rates of monotherapy in both groups might have been reflected to SPC preference, as prescriptions with sole SPC were limited to less than one-third of the prescriptions in both OPP (30.5%) and VOPP (26.6%). Combining two antihypertensives from different classes was reported to perform better in achieving optimal blood pressure levels, rather than increasing the dose of existing agent [11]. In this context, due to the benefits of more effective blood pressure reduction and better drug adherence, SPCs were recommended in ESC/ESH guidelines from 2013 [4]. The most recent guidelines even prioritized those combinations as the first-line option for the initiation of antihypertensive treatment in most groups [3]. Also, SPCs were associated with better cardiovascular outcomes in real-world studies, e.g. cardiovascular disease-related hospitalization risk reportedly being one-fourth of free combinations [12]. Thus, low preference of SPCs by general practitioners might be among the factors limiting the success of antihypertensive therapy in primary care. It should also be noted that as those guideline recommendations were not proposed specifically for primary care, their influence on the general practice might be delayed. In fact, a recent study from Bahrain reported limited implementation of updated guideline recommendations in primary care for hypertension treatment [13].

Thiazide diuretics were the top antihypertensive class in prescriptions with combination regimen, and slightly more common in OPP than VOPP (69.8% vs. 67.4%). Thiazides are deemed compatible to combine with many of the agents from other antihypertensive classes, which might explain thiazide-containing combination prescriptions being about 10-fold more than monotherapy counterparts in both groups [4]. Presence of ARBs or ACEIs in at least three-fifths in thiazide diuretic prescriptions was also noteworthy. Combination of diuretics with a renin-angiotensin-aldosterone system (RAAS) blocker might be regarded as rational, as it was reported to offer benefits especially in patients at risk of fluid retention and diagnosed with diabetes, heart failure, or renal impairment [14]. Among other diuretics, higher frequency of furosemide in VOPP (5.5% vs. 2.7% in OPP) was marked, with this drug being the third most common antihypertensive in this group. Loop diuretics, such as furosemide, are not pronounced among first-line options for hypertension treatment due to potential adverse outcomes. Aside from electrolyte disturbances and unwanted metabolic effects, furosemide might trigger volume depletion by increasing urination frequency, and potential rapid changes in extracellular fluid levels might also increase the risk for falls [15–17]. Furosemide might be chosen for the treatment of conditions such as heart failure and peripheral edema, which was reported to be around 3-to-5-fold in ≥80-year-old compared to under 60 [18]. A study from France in 2015 reported that 26.0% of patients over 80 were prescribed furosemide, with severe heart failure being the most common indication [19]. Nevertheless, while furosemide is indicated in heart failure treatment, the disproportionately high frequency in VOPP which was not observed for other heart failure drugs, e.g., beta-blockers and ACEIs, raises questions about the possibility of irrational prescribing behavior regarding this agent.

ARBs predominated over ACEIs in both OPP and VOPP. Although previous recommendations did not prioritize ARBs over ACEIs except select individuals mainly due to cost, ARBs were recently reported to have better safety profile [20, 21]. Preference of ARBs over ACEIs in our study period seems intriguing, which might be associated with Turkish reimbursement system compensating the costs without significant additional liability to the recipient. Despite known benefits when indicated, ACEIs and ARBs should be used cautiously in very elderly due to age-related decrease in glomerular filtration and subsequent increased risk of hyperkalemia [22, 23]. This potential issue might explain lower preference of both RAAS blockers in VOPP. Prescriptions containing both drug classes were also at a remarkable level. Combined use of ACEIs and ARBs was reported to increase the risk of hypotension, syncope, hyperkalemia, and renal failure without leading to any significant change in benefits; therefore, their combined use is not recommended [24]. ARBs were prescribed in 2.2% of ACEI prescriptions in OPP and 2.5% of those in VOPP, which is further concerning for very old patients due to higher potential of vulnerability. This may be related to insufficient knowledge of the physicians, as well as their repeat prescription habits. A study from the UK conducted in 2009–2015 reported that prescriptions including both RAAS blockers decreased 18.7% after an official warning from Medicine and Healthcare Products Regulatory Agency (MHRA) in 2014 [25]. Another study from the same country concluded that 77% of primary care prescriptions issued in 2011 were repeat prescriptions, and at least one repeat prescription was given to 43% of the population [26]. In order to avoid possible prescription errors, it might be suggested that repeat prescriptions should not be written without thorough evaluation of the patient, including regular drug use.

Hypertension is often accompanied by other diseases, mainly cardiovascular and metabolic disorders, and prevalence of comorbidities were reported to rise with age [6]. Aside from hypertension, beta-blockers established a wider range of indications in contrast to most other antihypertensives, including other cardiovascular diseases such as coronary artery disease and arrhythmias [27]. Beta-blockers were the second most frequently encountered antihypertensive class, and metoprolol led the antihypertensives in both OPP and VOPP. In fact, ischemic heart diseases were among the top five disorders accompanying hypertension in both groups, suggesting that frequent prescribing of beta-blockers might be associated with preference of agents covering multiple disorders for the patients with comorbidities during antihypertensive selection. Interestingly, beta-blockers were less frequent in VOPP, which were presumably issued to a population with higher number of comorbidities. This might be related to reportedly lower efficacy in cerebrovascular protection in elderly, tolerability issues, and lack of suitability for especially nonselective agents in conditions such as diabetes mellitus, asthma, and peripheral vascular diseases [28].

PIMs were more commonly encountered in VOPP, which were prescribed to a potentially frailer population. Similarly, a Chinese study reported higher rates of inpatient PIM prescribing to those over 80 compared to those aged 65–79 (58.2% vs. 43.4%), [29]. The top PIM class was cardiovascular drugs in both groups, of which were fully consisted of agents with antihypertensive effects. Most of the older antihypertensive drugs, including methyldopa and reserpine, are used in limited circumstances, or not used at all nowadays due to safety concerns, drug-drug interactions, and tolerability problems [30]. Alpha-1 receptor blockers are pronounced as viable options in certain circumstances rather than first-line antihypertensives according to the guidelines [3]. On the other hand, their use in elderly has been questionable due to safety concerns, mainly orthostatic hypotension and related outcomes such as falls and fractures [31]. The majority of potentially inappropriate antihypertensives in this study were alpha-1 blockers. Predilection for these agents might be associated with the need for treating benign prostate hyperplasia treatment symptoms in elderly men, as more than 50% over 60, and even as much as 90% of over 80 reportedly being affected [32]. In this context, alpha-1 blockers with higher prostate selectivity, e.g., silodosin and tamsulosin, might be deemed more appropriate options especially for this population susceptible to major adverse events [33].

NSAIDs, which were frequently utilized in both groups, were among the drugs of which potential inappropriateness could not be confirmed due to lack of follow-up since those agents being listed in Beers Criteria only for chronic use. A primary care study from İstanbul, in which 44.4% of participants were ≥65 years, reported that NSAIDs were commonly included in prescriptions with hypertension diagnosis, and they were prescribed irrationally [34]. Aside from gastrointestinal adverse outcomes and nephrotoxicity, NSAIDs were reported to trigger dysregulation of systolic blood pressure levels in hypertensive patients, resulting in reduced treatment success [35, 36]. Despite a sizable amount of those agents possibly being written for short-term utilization, them being available in approximately one-eighth of prescriptions, which entirely consisted of hypertensive recipients, might imply potentially irrational prescribing in a significant portion of those prescriptions in this context. Especially in VOPP, high prevalence of NSAIDs might be regarded as more concerning, since these drugs might further induce chronic kidney injury in individuals over 80, who might be under higher risk of compromised renal function [23, 37]. Thus, frequent utilization of NSAIDs in both groups point out the need to address a more rational approach for the utilization of drugs, especially the analgesics for the elderly in primary care.

The results of this study should be interpreted with its limitations. Since the study data only included the prescription information in the medical records, the diagnoses of the physicians were assumed as correct, and patient adherence to antihypertensives could not be evaluated. Also, blood pressure levels, physical examination data and laboratory values of the patients could not be obtained. In addition, due to the cross-sectional design of the study, clinical outcomes of the patients, such as effectiveness of the treatment, could not be assessed. All those issues complicated more profound evaluation of the association of individual treatment goals and prescribed antihypertensive regimen. Patient information was anonymized for ethical reasons, which prevented detecting previous prescriptions and prior medication history of the patients. Therefore, multiple prescriptions of several patients might have been possibly included. Moreover, this anonymized prescription record-based cross-sectional study did not involve follow-up of the patients, which made it unable to determine potential inappropriateness of some medications within the Beers list, e.g., NSAIDs and proton-pump inhibitors, clearly. Hence, we opted to present the drugs which are deemed potentially inappropriate only in long-term or first-line use separately in Table 5. It should be noted that a considerable portion of those agents might have actually been used appropriately. Additionally, medication history could influence the antihypertensive preference of the physicians, and our inability to access to that data precluded us to measure the respective effect. Finally, our data was limited to primary care; thus, we could not cover the prescribing practice in secondary or tertiary levels of healthcare. On the other hand, repeat prescriptions generated by primary care might indirectly provide information about the prescription habits of specialists working in more comprehensive hospitals.

In conclusion, this study revealed that prescribing preferences of primary care physicians to hypertensive old and very old patients showed slight differences. Almost half of prescriptions comprising monotherapy as antihypertensive regimen might imply hesitancy to prescribe combinations; thus, this could result in inadequate blood pressure control for a considerable portion of patients. Along with combining RAAS blockers, overuse of drugs which might pose risks to especially very elderly, such as furosemide and NSAIDs, implies irrational prescribing. The results of the study might provide guidance for the issues that need attention in utilization of antihypertensives and other drugs in the elderly and the very elderly. Concordance with the up-to-date evidence and thorough evaluation before drug selection might provide the basis for appropriate prescribing and optimal treatment.

## Supplementary materials

**Table S1:** The most common diagnoses accompanying hypertensive diseases (I10–I15) in the prescriptions of the study groups. Each of the most commonly prescribed five drugs in their respective category was presented in bold.

Diagnoses (ICD-10)	Total		Old patients		Very old patients	
	Rank	n (%)	Rank	n (%)	Rank	n (%)
**Diseases of esophagus, stomach, and duodenum (K20–K31)**	**1**	132,072 (11.8)	**1**	98,665 (11.8)	**1**	33,407 (11.6)
**Diabetes mellitus (E10–E14)**	**2**	99,166 (8.8)	**2**	82,274 (9.9)	**3**	16,892 (5.9)
**Soft tissue disorders (M60–M79)**	**3**	79,471 (7.1)	**3**	61,223 (7.3)	**2**	18,248 (6.3)
**Metabolic disorders (E70–E90)**	**4**	51,237 (4.6)	**4**	41,675 (5.0)	7	9562 (3.3)
**Ischaemic heart diseases (I20–I25)**	**5**	51,087 (4.5)	**5**	37,447 (4.5)	**4**	13,640 (4.7)
Arthropathies (M00–M25)	6	42,327 (3.8)	6	31,326 (3.8)	6	11,001 (3.8)
Other nutritional deficiencies (E50–E64)	7	41,587 (3.7)	7	30,258 (3.6)	**5**	11,329 (3.9)
Persons encountering health services for examination and investigation (Z00–Z13)	8	39,049 (3.5)	8	30,021 (3.6)	9	9028 (3.1)
Dermatitis and eczema (L20–L30)	9	33,444 (3.0)	9	24,551 (2.9)	10	8893 (3.1)
Chronic lower respiratory diseases (J40–J47)	10	32,630 (2.9)	11	23,413 (2.8)	8	9217 (3.2)
General symptoms and signs (R50–R69)	11	31,944 (2.8)	10	24,039 (2.9)	12	7905 (2.7)
Mood [affective] disorders (F30–F39)	12	28,513 (2.5)	13	20,024 (2.4)	11	8489 (2.9)
Acute upper respiratory infections (J00–J06)	13	26,427 (2.3)	12	20,886 (2.5)	15	5541 (1.9)
Dorsopathies (M40–M54)	14	24,871 (2.2)	15	19,116 (2.3)	14	5755 (2.0)
Disorders of thyroid gland (E00–E07)	15	24,250 (2.2)	14	19,576 (2.3)	21	4674 (1.6)
Mycoses (B35–B49)	16	23,059 (2.1)	16	17,928 (2.1)	19	5131 (1.8)
Other diseases of upper respiratory tract (J30–J39)	17	20,894 (1.9)	17	16,375 (2.0)	23	4519 (1.6)
Other diseases of intestines (K55–K64)	18	20,168 (1.8)	19	13,233 (1.6)	13	6935 (2.5)
Diseases of male genital organs (N40–N51)	19	19,278 (1.7)	18	14,066 (1.7)	17	5212 (1.8)
Neurotic, stress-related and somatoform disorders (F40–F48)	20	17,774 (1.6)	20	12,637 (1.5)	18	5137 (1.8)
Symptoms and signs involving cognition, perception, emotional state and behavior (R40–R46)	21	15,665 (1.4)	21	10,339 (1.2)	16	5326 (1.8)
Other forms of heart disease (I30–I52)	22	14,462 (1.3)	22	9745 (1.2)	20	4717 (1.6)
Osteopathies and chondropathies (M80–M94)	23	13,782 (1.2)	24	9126 (1.1)	22	4656 (1.6)
Nutritional anemia (D50–D53)	24	13,271 (1.2)	23	9419 (1.1)	27	3852 (1.3)
Other diseases of urinary system (N30–N39)	25	12,910 (1.1)	26	8646 (1.0)	24	4264 (1.5)
Other and unspecified effects of external causes (T66–T78)	26	12,219 (1.1)	25	8894 (1.1)	29	3325 (1.2)
Diseases of veins, lymphatic vessels and lymph nodes, not elsewhere classified (I80–I89)	27	11,279 (1.0)	27	8198 (1.0)	30	3081 (1.1)
Episodic and paroxysmal disorders (G40–G47)	28	10,824 (1.0)	28	7472 (0.9)	28	3352 (1.2)
Behavioral and emotional disorders with onset usually occurring in childhood and adolescence (F90–F98)	29	7135 (0.6)	29	7135 (0.9)	32	2916 (1.0)
Polyneuropathies and other disorders of the peripheral nervous system (G60–G64)	30	7042 (0.6)	30	7042 (0.8)	36	2083 (0.7)
Others		165,527 (14.7)		109,971 (13.2)		50,557 (17.5)
**Total**		**1,123,364 (100.0)**		**834,720 (100.0)**		**288,644 (100.0)**

* Diagnoses not shown for the very old age group were “Other degenerative diseases of the nervous system (G30–G32)” (n = 4174, 1.4%, ranked 25th) and “Cerebrovascular diseases (I60–I69)” (n = 4138, 1.4%, ranked 26th).

**Table S2:** The distribution of the most commonly encountered drugs in hypertension prescriptions in the study groups.*

Drugs (ATC-5)	Total		Old patients		Very old patients	
	Rank	n (%)	Rank	n (%)	Rank	n (%)
**Acetylsalicylic acid (B01AC06)**	**1**	124,623 (5.8)	**1**	96,225 (5.9)	**1**	28,398 (5.4)
**Metoprolol (C07AB02)**	**2**	102,078 (4.8)	**2**	78,308 (4.8)	**2**	23,770 (4.5)
**Amlodipine (C08CA01)**	**3**	64,783 (3.0)	**3**	48,822 (3.0)	**3**	15,961 (3.0)
**Metformin (A10BA02)**	**4**	53,856 (2.5)	**4**	45,902 (2.8)	10	7954 (1.5)
**Diclofenac (M01AB05)** **	**5**	42,165 (2.0)	**5**	32,731 (2.0)	**4**	9434 (1.8)
Pantoprazole (A02BC02)	6	36,971 (1.7)	8	27,650 (1.7)	**5**	9321 (1.8)
Atorvastatin (C10AA05)	7	36,570 (1.7)	6	29,800 (1.8)	13	6770 (1.3)
Valsartan and diuretics (C09DA03)	8	35,116 (1.6)	9	27,394 (1.7)	11	7722 (1.5)
Other nontherapeutic auxiliary products (V07AY)	9	34,918 (1.6)	7	29,555 (1.8)	18	5363 (1.0)
Lansoprazole (A02BC03)	10	33,644 (1.6)	10	24,777 (1.5)	7	8867 (1.7)
Paracetamol (N02BE01)	11	32,072 (1.5)	12	23,148 (1.4)	6	8924 (1.7)
Vitamin B1 in comb. with vitamin B6 and/or vitamin B12 (A11DB)	12	31,260 (1.5)	13	22,519 (1.4)	8	8741 (1.7)
Candesartan and diuretics (C09DA06)	13	29,598 (1.4)	11	23,651 (1.5)	15	5947 (1.1)
Carvedilol (C07AG02)	14	26,405 (1.2)	16	19,495 (1.2)	12	6910 (1.3)
Ramipril (C09AA05)	15	26,343 (1.2)	15	20,408 (1.3)	16	5935 (1.1)
Levothyroxine sodium (H03AA01)	16	25,971 (1.2)	14	21,377 (1.3)	24	4594 (0.9)
Other cold preparations (R05X)	17	23,873 (1.1)	17	19,414 (1.2)	27	4459 (0.8)
Furosemide (C03CA01)	18	22,816 (1.1)	25	14,076 (0.9)	9	8740 (1.7)
Gliclazide (A10BB09)	19	21,807 (1.0)	18	17,588 (1.1)	33	4219 (0.8)
Clopidogrel (B01AC04)	20	21,469 (1.0)	20	16,130 (1.0)	19	5339 (1.0)
Escitalopram (N06AB10)	21	21,358 (1.0)	22	15,353 (0.9)	14	6005 (1.1)
Nebivolol (C07AB12)	22	20,796 (1.0)	19	17,076 (1.1)	38	3720 (0.7)
Perindopril and diuretics (C09BA04)	23	20,205 (0.9)	21	15,825 (1.0)	28	4380 (0.8)
Esomeprazole (A02BC05)	24	18,624 (0.9)	24	14,338 (0.9)	32	4286 (0.8)
Ramipril and diuretics (C09BA05)	25	18,254 (0.9)	23	14,362 (0.9)	35	3892 (0.7)
Lercanidipine (C08CA13)	26	18,126 (0.8)	26	13,760 (0.8)	29	4366 (0.8)
Losartan and diuretics (C09DA01)	27	17,842 (0.8)	27	13,642 (0.8)	34	4200 (0.8)
Doxazosin (C02CA04) **	28	17,564 (0.8)	29	13,226 (0.8)	30	4338 (0.8)
Imidazoles/triazoles in comb. with corticosteroids (D01AC20)	29	17,148 (0.8)	28	13,315 (0.8)	37	3833 (0.7)
Indapamide (C03BA11)	30	16,991 (0.8)	31	12,240 (0.8)	22	4751 (0.9)
Others		1,134,243 (52.8)		838,924 (51.7)		295,319 (56.1)
**Total**		**2,147,489 (100)**		**1,621,031 (100)**		**526,458 (100)**

Each of the most commonly prescribed five drugs in their respective category was presented in bold.

ATC-5 denotes the code of the particular drug (fifth level) of the Anatomical Therapeutic Chemical classification of drugs.

* The drug ranked 30th in OPP was dexketoprofen (12,424, 0.8%), whereas the drugs not shown above for VOPP and their rankings were as follows: trimetazidine (n = 5404, 1.0%, ranked 17th), topical diclofenac (n = 5043, 1.0%, ranked 20th), isosorbide mononitrate (n = 4859, 0.9%, ranked 21st), piracetam (n = 4727, 0.9%, ranked 23rd), betahistine (4492, 0.9%, ranked 25th), paracetamol combinations (n = 4477, 0.9%, ranked 27th).

** Drugs classified as “potentially inappropriate medications” according to Beers Criteria.

## Figures and Tables

**Figure:**
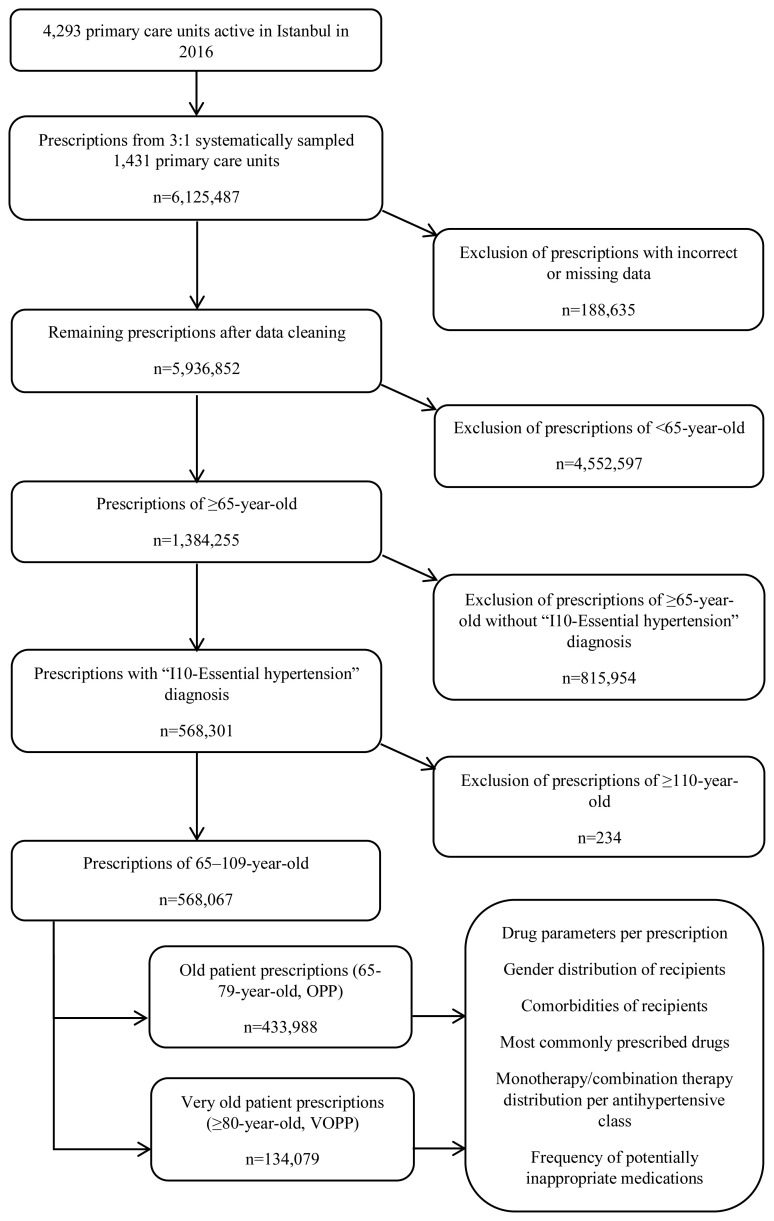
Flow chart of the study.

**Table 1:** Demographic and drug utilization characteristics in the prescriptions in the study population.

	Total	Old patients	Very old patients
Prescriptions, n (%)	568,067 (100.0)	433,988 (76.4)	134,079 (23.6)
Female, n (%)*	350,908 (61.8)	260,792 (60.1)	90,116 (67.2)
Age, years, mean ± SD	74.4 ± 7.1	71.2 ± 4.2	84.7 ± 3.9
Encounters with multiple diagnoses, n (%)*	439,264 (77.3)	333,486 (76.9)	105,778 (78.9)
Drug box per encounter, mean ± SD*	11.4 ± 10.0	11.0 ± 8.7	12.6 ± 13.4
Drug item per encounter, mean ± SD*	3.8 ± 2.2	3.7 ± 2.2	3.9 ± 2.3
Antihypertensive drugs per encounter, mean ± SD*	1.2 ± 0.7	1.2 ± 0.7	1.2 ± 0.7
Encounters with ≥1 antihypertensive drug(s), n (%)*	509,713 (89.7)	390,319 (89.9)	119,394 (89.0)
Encounters with only single antihypertensive agent, n (%)*	223,183 (43.8)	169,080 (43.3)	54,103 (45.3)
Encounters with only one single-pill antihypertensive combination, n (%)*	150,766 (29.6)	119,075 (30.5)	31,691 (26.6)
Encounters with multiple-pill antihypertensive therapy#, n (%)*	135,764 (26.6)	102,164 (26.2)	33,600 (28.1)

SD: standard deviation.

# Includes encounters with more than one single-pill combination, or with free combinations.

* p < 0.001 for pairwise comparisons between prescriptions of old and very old age groups.

**Table 2:** Distribution of the most commonly prescribed antihypertensive drugs in the study groups.*

Drugs (ATC-5)	Old patients		Very old patients	
	n (%)	Rank	n (%)	Rank
**Metoprolol (C07AB02)**	**78,308 (15.3)**	**1**	**23,770 (14.8)**	**1**
**Amlodipine (C08CA01)**	**48,822 (9.5)**	**2**	**15,961 (10.0)**	**2**
**Valsartan and diuretics (C09DA03)**	**27,394 (5.4)**	**3**	**7722 (4.8)**	**4**
**Candesartan and diuretics (C09DA06)**	**23,651 (4.6)**	**4**	5947 (3.7)	6
**Ramipril (C09AA05)**	**20,408 (4.0)**	**5**	5935 (3.7)	7
**Carvedilol (C07AG02)**	19,495 (3.8)	6	**6910 (4.3)**	**5**
Nebivolol (C07AB12)	17,076 (3.3)	7	3720 (2.3)	15
Perindopril and diuretics (C09BA04)	15,825 (3.1)	8	4380 (2.7)	9
Ramipril and diuretics (C09BA05)	14,362 (2.8)	9	3892 (2.5)	14
**Furosemide (C03CA01)**	14,076 (2.7)	10	**8740 (5.5)**	**3**
Lercanidipine (C08CA13)	13,760 (2.7)	11	4366 (2.7)	10
Losartan and diuretics (C09DA01)	13,642 (2.7)	12	4200 (2.6)	13
Doxazosin (C02CA04)**	13,226 (2.6)	13	4338 (2.7)	11
İndapamide (C03BA11)	12,240 (2.4)	14	4751 (3.0)	8
Irbesartan and diuretics (C09DA04)	11,756 (2.3)	15	3374 (2.1)	17
Nifedipine (C08CA05)	11,175 (2.2)	16	4301 (2.7)	12
Valsartan and amlodipine (C09DB01)	9975 (1.9)	17	2172 (1.4)	23
Perindopril and amlodipine (C09BB04)	9349 (1.8)	18	2106 (1.3)	26
Telmisartan and diuretics (C09DA07)	9123 (1.8)	19	2473 (1.5)	18
Olmesartan and diuretics (C09DA08)	8632 (1.7)	20	2129 (1.3)	25
Perindopril (C09AA04)	8619 (1.7)	21	2344 (1.5)	19
Diltiazem (C08DB01)	7979 (1.6)	22	3673 (2.3)	16
Bisoprolol (C07AB07)	7875 (1.5)	23	2154 (1.3)	24
Lisinopril and diuretics (C09BA03)	7194 (1.4)	24	2292 (1.4)	20
Valsartan (C09CA03)	6446 (1.3)	25	2016 (1.3)	27
Candesartan (C09CA06)	5705 (1.1)	26	1440 (0.9)	30
Trandolapril and verapamil (C09BB10)	5084 (1.0)	27	1292 (0.8)	31
HCTZ and potassium-sparing agents (C03EA01)	4916 (1.0)	28	2252 (1.4)	21
Spironolactone (C03DA01)	4880 (1.0)	29	2177 (1.4)	22
Losartan (C09CA01)	4737 (0.9)	30	1618 (1.0)	29
Others	56,233 (11.0)		17,457 (10.9)	
Total	511,963 (100.0)		160,300 (100.0)	

Each of the most commonly prescribed five drugs in their respective category was presented in bold. ATC-5 denotes the code of the particular drug (fifth level) of the Anatomical Therapeutic Chemical classification of drugs.

* 28th drug in the very old group was captopril (C09AA01) (1690; 1.1%). HCTZ, hydrochlorothiazide.

** Classified as ‘potentially inappropriate medications’ according to Beers Criteria.

**Table 3:** Characteristics of main antihypertensive drug-containing prescriptions in the study groups.

	Thiazide diuretic prescriptions (n = 210,746)		Beta blocker prescriptions (n = 171,282)		ARB prescriptions (n = 166,715)		Calcium antagonist prescriptions (n = 154,726)		ACEI prescriptions (n = 136,389)	
	Old patients	Very old patients	Old patients	Very old patients	Old patients	Very old patients	Old patients	Very old patients	Old patients	Very old patients
Total (n = 509,713)	**41.8**	**39.9**	**33.9**	**32.7**	**33.6**	**29.9**	**30.1**	**31.3**	**27.1**	**25.6**
Monotherapy (n = 223,183)	**5.2**	**6.7**	**37.4**	**33.1**	**9.5**	**8.1**	**27.7**	**30.1**	**14.1**	**12.8**
Combined therapy (n = 286,530)	**69.8**	**67.4**	**31.1**	**32.5**	**52.0**	**47.9**	31.9	32.2	**37.1**	**36.3**
with thiazide diuretics	1.3	1.6	51.9	49.0	84.7	84.9	35.9	35.5	63.8	65.3
with beta blockers	23.1	23.6	1.6	2.0	24.2	24.4	31.2	33.1	24.2	26.3
with ARBs	63.1	60.4	40.3	35.9	0.3	0.3	40.9	36.3	2.2	2.5
with calcium antagonists	16.4	17.0	31.9	32.9	25.1	24.4	1.9	2.8	34.0	32.7
with ACEIs	33.9	35.2	32.3	29.4	1.6	1.9	39.6	36.8	1.3	1.4
with other antihypertensives	4.3	4.0	14.0	20.2	4.6	6.7	8.9	13.4	5.9	9.0

p < 0.001 for all total, monotherapy, and combined therapy stratifications in main antihypertensive drug-containing prescriptions, which were presented in bold (except the percentage of combinations of calcium antagonists in those belonging to old vs. very old patients).

**Table 4:** Comparison of potentially inappropriate medications per Beers Criteria in old vs. very old patient prescriptions.

	Potentially inappropriate drugs per 10,000 prescriptions				Drug classes in all prescriptions by Beers category						
	Old patients	Very old patients	Total	n	Old patients		Very old patients		Total		p-value
					%	n	%	n	%		
**Anticholinergic drugs** *	92.3	173.0	111.3	Potentially inappropriate	4004	16.0	2319	25.7	6323	18.6	<0.001
				Appropriate	21,048	84.0	6700	74.3	27,748	81.4	
				Total	25,052	100.0	9019	100.0	34,071	100.0	
**Antithrombotic agents**	21.8	44.2	27.1	Potentially inappropriate	946	0.8	593	1.5	1539	1.0	<0.001
				Appropriate	122,143	99.2	38,020	98.5	160,163	99.0	
				Total	123,089	100.0	38,613	100.0	161,702	100.0	
**Cardiovascular drugs**	263.9	283.4	268.5	Potentially inappropriate	11,455	1.9	3800	2.0	15,255	2.0	0.004
				Appropriate	578,237	98.1	181,557	98.0	759,794	98.0	
				Total	589,692	100.0	185,357	100.0	775,049	100.0	
**Antidepressants**	104.4	100.8	103.6	Potentially inappropriate	4533	11.2	1352	8.8	5885	10.5	<0.001
				Appropriate	36,138	88.8	14,106	91.2	50,244	89.5	
				Total	40,671	100.0	15,458	100.0	56,129	100.0	
**Antipsychotics**	50.6	158.0	76.0	Potentially inappropriate	2197	45.4	2119	51.4	4316	48.2	<0.001
				Appropriate	2636	54.6	1998	48.6	4634	51.8	
				Total	4833	100.0	4117	100.0	8950	100.0	
**Barbiturates**	<0.1	0.0	<0.1	Potentially inappropriate	2	0.5	0	-	2	0.3	-
				Appropriate	384	99.5	164	100.0	548	99.7	
				Total	386	100.0	164	100.0	550	100.0	
**Benzodiazepines** **	8.3	17.2	10.5	Potentially inappropriate	362	82.3	232	86.8	594	84.1	0.09
				Appropriate	78	17.7	34	13.2	112	15.9	
				Total	440	100.0	266	100.0	706	100.0	
**Endocrine system drugs**	1.3	1.4	1.3	Potentially inappropriate	56	<0.1	19	0.1	75	<0.1	0.04
				Appropriate	134,711	>99.9	26,110	99.9	160,821	>99.9	
				Total	134,767	100.0	26,129	100.0	160,896	100.0	
**Gastric motility drugs**	18.8	27.5	20.8	Potentially inappropriate	814	35.5	369	34.6	1183	35.2	0.64
				Appropriate	1481	64.5	698	65.4	2179	64.8	
				Total	2295	100.0	1067	100.0	3362	100.0	
**Pain medications**	25.6	33.6	27.4	Potentially inappropriate	1109	1.0	450	1.2	1559	1.1	0.01
				Appropriate	105,666	99.0	37,024	98.8	142,690	98.9	
				Total	106,775	100.0	37,474	100.0	144,249	100.0	
**Skeletal muscle relaxants**	102.0	97.9	101.0	Potentially inappropriate	4426	19.1	1312	21.7	5738	19.6	<0.001
				Appropriate	18,787	80.9	4731	78.3	23,518	80.4	
				Total	23,213	100.0	6043	100.0	29,256	100.0	
**Genitourinary drugs**	1.3	0.5	1.1	Potentially inappropriate	56	0.3	7	0.1	63	0.2	<0.001
				Appropriate	20,496	99.7	8950	99.9	29,446	99.8	
				Total	20,552	100.0	8957	100.0	29,509	100.0	
**Total**	690.4	937.7	748.7	Potentially inappropriate	29,960	2.8	12,572	3.8	42,532	3.0	<0.001
				Appropriate	1,041,805	97.2	320,092	96.2	1,361,897	97.0	
				Total	1,071,765	100.0	332,664	100.0	1,404,429	100.0	

* Include first-generation antihistamines.

** Include short- and long-acting benzodiazepines.

**Table 5:** Distribution of potentially inappropriate drugs with no follow-up data.

	Potentially inappropriate medications						Recommendation in Beers Criteria
	Old patients		Very old patients		Total		
	n	Per 10,000 prescriptions	n	Per 10,000 prescriptions	n	Per 10,000 prescriptions	
**Nitrofurantoin**	1094	25.2	522	38.9	1616	28.4	Avoid in individuals with creatinine clearance <30 mL/min or for long-term suppression of bacteria
**Digoxin**	2201	50.7	1,250	93.2	3451	60.7	Avoid as first-line therapy for atrial fibrillation Avoid as first-line therapy for heart failure If used for atrial fibrillation or heart failure, avoid dosages >0.125 mg/d
**Amiodarone**	1237	28.5	508	37.9	1745	30.7	Avoid amiodarone as first-line therapy for atrial fibrillation unless patient has heart failure or substantial left ventricular hypertrophy
**Antipsychotics** *	37	0.9	26	1.9	63	1.1	Avoid, except for short-term use as antiemetic during chemotherapy
**Proton pump inhibitors**	77,096	1776.4	25,538	1904.7	102,634	1806.7	Avoid scheduled use for >8 weeks unless for high-risk patients (e.g., oral corticosteroids or chronic NSAID use), erosive esophagitis, Barrett’s esophagitis, pathological hypersecretory condition, or demonstrated need for maintenance treatment (e.g., due to failure of drug discontinuation trial or H2 blockers)
**Nonsteroidal antiinflammatory drugs**	54,809	1262.9	15,422	1150.2	70,231	1236.3	Avoid chronic use, unless other alternatives are not effective and patient can take gastroprotective agent (proton-pump inhibitor or misoprostol)
**Total**	136,474	3144.6	43,266	3226.8	179,740	3163.9	

* includes antipsychotics present in prescriptions with “Nausea and vomiting (R11)” diagnosis according to 10th Revision of International Classification of Diseases (ICD-10).

## Footnotes

1 TURKSTAT - Turkish Statistical Institute (2017). The Results of Address Based Population Registration System, 2016 [online]. Website https://data.tuik.gov.tr/Bulten/Index?p=The-Results-of-Address-Based-Population-Registration-System-2016-24638 [accessed 29 August 2022].

2 TURKSTAT - Turkish Statistical Institute (2019). Life Tables, 2016-2018 [online]. Website https://data.tuik.gov.tr/Bulten/Index?p=30712&-dil=2 [accessed 04 November 2022].
